# Influence of Storage Temperature on Radiochemical Purity of ^99m^Tc-Radiopharmaceuticals

**DOI:** 10.3390/molecules23030661

**Published:** 2018-03-15

**Authors:** Licia Uccelli, Alessandra Boschi, Petra Martini, Corrado Cittanti, Stefania Bertelli, Doretta Bortolotti, Elena Govoni, Luca Lodi, Simona Romani, Samanta Zaccaria, Elisa Zappaterra, Donatella Farina, Carlotta Rizzo, Melchiore Giganti, Mirco Bartolomei

**Affiliations:** 1 Morphology, Surgery and Experimental Medicine Department, University of Ferrara, Via L. Borsari, 46, 44121 Ferrara (FE), Italy; alessandra.boschi@unife.it (A.B.); corrado.cittanti@unife.it (C.C.); carlotta.rizzo@student.unife.it (C.R.); ggm@unife.it (M.G.); 2 Nuclear Medicine Unit, University Hospital, Via Aldo Moro, 8, 44124 Ferrara (FE), Italy; s.bertelli@ospfe.it (S.B.); d.bortolotti@ospfe.it (D.B.); e.govoni@ospfe.it (E.G.); l.lodi@ospfe.it (L.L.); s.romani@ospfe.it (S.R.); s.zaccaria@ospfe.it (S.Z.); e.zappaterra@ospfe.it (E.Z.); d.farina@ospfe.it (D.F.); m.bartolomei@ospfe.it (M.B.); 3 Physics and Heart Science Department, University of Ferrara, Via Giuseppe Saragat, 1, 44122 Ferrara (FE), Italy; petra.martini@unife.it; 4 Legnaro National Laboratories, Italian National Institute for Nuclear Physics (LNL-INFN), Viale dell’Università, 2, 35020 Legnaro (PD), Italy

**Keywords:** ^99m^Tc-radiopharmaceuticals, radiochemical purity, radiopharmaceuticals quality control

## Abstract

The influence of effective room temperature on the radiochemical purity of ^99m^Tc-radiopharmaceuticals was reported. This study was born from the observation that in the isolators used for the preparation of the ^99m^Tc-radiopharmaceuticals the temperatures can be higher than those reported in the commercial illustrative leaflets of the kits. This is due, in particular, to the small size of the work area, the presence of instruments for heating, the continuous activation of air filtration, in addition to the fact that the environment of the isolator used for the ^99m^Tc-radiopharmaceuticals preparation and storage is completely isolated and not conditioned. A total of 244 ^99m^Tc-radiopharmaceutical preparations (seven different types) have been tested and the radiochemical purity was checked at the end of preparation and until the expiry time. Moreover, we found that the mean temperature into the isolator was significantly higher than 25 °C, the temperature, in general, required for the preparation and storage of ^99m^Tc-radiopharmaceuticals. Results confirmed the radiochemical stability of radiopharmaceutical products. However, as required in the field of quality assurance, the impact that different conditions than those required by the manufacturer on the radiopharmaceuticals quality have to be verified before human administration.

## 1. Introduction

Radiopharmaceuticals labelled with technetium-99m (^99m^Tc) have been, and still are, by far the most used conventional agents for single photon emission computed tomography (SPECT). These radiopharmaceuticals are prepared directly in hospital sites, using commercial lyophilized kit formulations, by introducing the ^99^Mo/^99m^Tc generator-eluted ^99m^Tc-pertechnetate into the kit and following the manufacturer’s instruction contained in the package leaflet. The instructions concern not only the reconstitution procedure of the kit formulation, but also the storage conditions and the associated quality control procedures. Environmental factors, such as temperature, humidity, and/or light, intrinsic factors, such as chemical-physical properties of the reagents and excipients present in the kit formulations, pharmaceutical composition, quality of the generator eluted [^99m^Tc]NaTcO_4_, etc., could compromise the radiochemical purity of technetium-99m radiopharmaceuticals [1,2,3,4]. In particular, these factors could cause the formation of chemical impurities inducing phenomena such as hydrolysis and oxidation-reduction reactions. Among the impurities, residual ^99m^Tc-pertechnetate, as consequence of non-reduction, and the reduced hydrolyzed technetium-99m [^99m^Tc]TcO_2_, due to the pertechnetate reduction, but not the complexation of the technetium-99m [5], lead to consequent radiochemical purity (RCP) loss of the final radiopharmaceutical. The effect of many listed factors on RCP of some ^99m^Tc-radiopharmaceuticals have been investigated in the past, e.g., the technetium-99 presence excess in solution [2]; the humidity influence on radiochemical purity of [^99m^Tc]Tc-ECD and [^99m^Tc]Tc-MIBI [1]; the saline storage condition, used for the preparation of [^99m^Tc]Tc-HMPAO [6]; and the effect of increased temperature on the labelling efficiency of some ^99m^Tc-radiopharmaceutilcals [7]. In particular, Maksin et al. exposed some compounds, such as pyrophosphate (PyP), dicarboxypropane diphosphonate (DPD), dimercaptosuccinate (DMS), and Sn-colloid, at 37 °C for different time intervals before labelling with ^99m^Tc-pertechnetate. They found that, only for ^99m^Tc-Sn-colloids, the increased temperature exposure had no effect on the expected labelling yield in the routine quality control. Taking into account these results and in particular, considering that in our nuclear medicine hospital unit the preparation of ^99m^Tc-radiopharmaceuticals takes place inside a not conditioned isolator and, therefore, the temperature could be significantly higher than required by manufacturer, we decided to investigate the effect of the temperature on the radiochemical purity of radiopharmaceuticals at the end of the preparation and during the storage before injection. In fact, while the temperature required by manufacturers for the production of ^99m^Tc-radiopharmaceuticals is variable, the radiolabelled kits storage temperature value has to be <25 °C. Furthermore, the cited works [6,7] analyzed different aspects from this that we have investigated.

The aim of this work was to investigate the effect of storage temperature on the radiochemical purity of Ceretec (GE Healthcare, Milan, Italy), MAASOL (GE Healthcare, Milan, Italy), Nanocoll (GE Healthcare, Milan, Italy), Renocis (IBA molecular Italy S.r.l., Milan, Italy), Technemibi (Mallinckrodt, Petten, The Netherlands), Technescan HDP (Mallinckrodt, Petten, The Netherlands) and Technescan MAG3 (Mallinckrodt, Petten, The Netherlands).

## 2. Materials and Methods

All materials used and the methods followed are described in the manufacturer's instructions. These instructions represent the reference for operators in the production and quality control of radiopharmaceuticals. All tools and equipment described in the following sections were initially qualified (installation qualification, operational qualification, and performance qualification) and periodically re-qualified. 

### 2.1. ^99m^Tc-Radiopharmaceuticals Preparation

**Figure 1:**
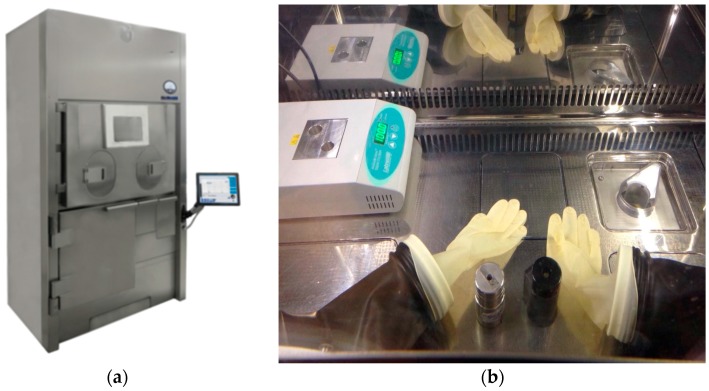
Murphil-Tc (MecMurphil): the isolator (**a**) and the work area inside the isolator (**b**).

^99^Mo/^99m^Tc Drytec generators (25 GBq of ^99^Mo) were purchased by GE (GE Healthcare S.r.l, Milan, Italy). Generator elution and ^99m^Tc-radiopharmaceuticals synthesis from kit formulation were conducted into a class A laminar flow isolator (Murphil-Tc, MecMurphil S.r.l, Ferrara, Italy, Figure 1), placed in a class D room. 

Freeze-dried kits (Table 1) have been stored until their use in a temperature-controlled refrigerator (Medical Proget, KW control, marketed by MecMurphil S.r.l, Ferrara, Italy). 

**Figure 2:**
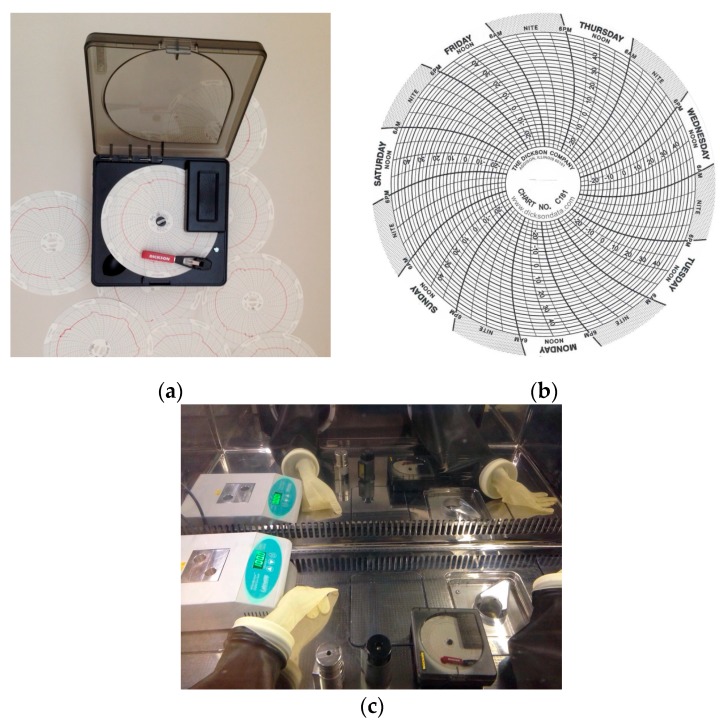
Graphic recorder (**a**,**b**) diagram disks (Dickson); and (**c**) recorder position in the work area.

Kit reconstitution was performed according to the methods described in the package insert included within the commercial kits. In order to evaluate radiochemical purity in the worst case, the volumes and the activities used during the reconstitution of the kit were the maximum possible. When required by the synthesis process, e.g., in the [^99m^Tc]Tc-MAG3 and [^99m^Tc]Tc-MIBI production (Table 2), a digital dry heater (AccuBlockTM, LabNet, purchased by MecMurphil S.r.l, Ferrara, Italy) was used. After labelling with ^99m^Tc-pertechnetate, the radiopharmaceuticals solutions were stored inside the isolator until the complete fractionation. Continuous temperature monitoring inside the isolator was performed using calibrated graphic recorder KT621 (Dickson, provided by the University Hospital of Ferrara). The temperature was recorded on a 101 mm diameter paper disc using a red pen (Figure 2). 

A preliminary temperature registration, in order to ascertain the temperature fluctuation, was made for 24 h/7 days for three weeks inside the isolator (hot cell closed): on the outer edge of the paper disc are indicated the different days of the week divided into three hours-groups; the temperatures, printed on the disk in the range from −30 °C to +50 °C, are then detected by a graduated scale with a precision of ±2.5 °C. Every Friday, the paper disk was replaced and the data were collected and registered into datasheets. The same temperature control has been continuously performed during all the radiochemical purity three months monitoring.

### 2.2. ^99m^Tc-Radiopharmaceuticals Radiochemical Purity Assessment

The radiochemical purity (RCP) of radiopharmaceuticals was evaluated immediately after the preparation (time = 0), in the middle and at the end of the expiry time indicated by the manufacturer (Table 1). The radiochemical purity was measured using methods specified by manufacturer, with the exception of Technescan (Mallinckrodt) for which the following chromatographic system was used [8]: mobile phase, 54/45/1 (physiological/methanol/glacial acetic acid, *v*/*v*/*v*) and stationary phase, RP-18 plates (Merck, Serono, Roma, Italy). The radioactivity distribution’s determination was performed by a scanning radio-chromatography detection system for thin layer chromatography (Cyclone instrument equipped with a phosphor imaging screen and an OptiQuant image analysis software; PerkinElmer, Waltham, MA, USA). The solvents were obtained from Sigma Aldrich (Sigma-Aldrich, Milan, Italy); only methanol was obtained from VWR (VWR International, Fontanay sous Bois, France); MilliQ (18.2 MΩ) water was obtained from a Direct Q^®^ system (Millipore, Darmstadt, Germany); physiological solution Fresenius KABI was obtained from Medexitalia (Medexitalia, Roma, Italy). All mobile phases used for quality control of radiopharmaceuticals were stored in a controlled and ventilated refrigerator (Antiscintilla 400 ECT-F-TOUCH, Fiocchetti, marketed by MecMurphil S.r.l, Ferrara, Italy); the stationary phases were stored in a special dehumidified container. ITLC-SG and ITLC-SA plates were obtained from Agilent Technology (Folsom, CA, USA); Whatman n.1 (Merck, Serono, Roma, Italy). Table 2 summarizes all of the experimental variables.

## 3. Results and Discussion

The illustrative leaflets of the commercial kits used for the preparation of ^99m^Tc-radiopharmaceuticals, set out in detail all the preparations conditions, the quality controls to be executed, the values of the radiochemical purity and storage conditions, such as the temperature to which the radiopharmaceutical must be preserved before injection.

The small size of the work area, the presence of instruments for heating, the continuous activation of air filtration, the internal lighting, in addition to the fact that the environment of the isolator, used for the preparation and holding of ^99m^Tc-radiopharmaceuticals, is completely isolated and not conditioned (Figure 1), could affect the temperature in the work area. At the moment, to our knowledge, there are no conditioning-equipped isolators, for nuclear medicine, available on the market even though the nuclear medicine community has chorally underlined this problem to the hot cells producers.

These potential discrepancies of temperatures and its influence on the radiochemical purity of radiopharmaceuticals have been deeply investigated in this work. The first objective was to verify the real temperature inside the isolator, with a calibrated instrument (Figure 1). The temperature required by manufacturers for the production of radiopharmaceuticals is variable, but the storage value must be <25 °C. Data were collected for three months (7 days/24 h) and the results are summarized in Table 3. The data collected through the graphical temperature recorder were firstly transcribed and subdivided by time slots in an Excel sheet. From these data, it was possible to determine the temperature average values and error, on different days of the week, divided by time slots. 

The results show a significant difference in temperatures compared to the reference standard (<25 °C). In the afternoon the instrument used to heat is off, but, despite this, the temperature, on average, results higher than 25 °C; this fact can be explained, most likely, by the operation of the isolator ventilation systems, which causes air heating. Establishing that the internal temperature of the isolator differed from that required for storage, we evaluated how much this temperature difference could affect the RCP of radiopharmaceuticals during the storage period, which also includes the fractionation of the preparation to be administered to the patients. A total of 244 ^99m^Tc-radiopharmaceutical preparations (seven different types), performed in our nuclear medicine unit, have been tested in this work. Table 4 shows the RCP values of the preparations, reported as the mean and standard deviation of the individual values found during the three months of the test run. Along with these values, the temperature mean values detected during the RCP determination were added.

The results show that the temperatures are well above the reference standards but the RCP values are always higher than those established by manufacturers. The overall analysis of the results showed no anomalies with respect to the radiopharmaceutical quality of the products intended for administration to the patients at the time of preparation or at the limit of their stability. Moreover, all “media fill” tests confirmed the sterility of the radiopharmaceutical products. 

## 4. Conclusions

The radiochemical purity of radiopharmaceuticals plays a key role in the protection of the patient to which the minimum undesirable exposure must be ensured. The evaluation of the impact that different conditions may have on the radiopharmaceuticals quality, it is crucial. The results of this work confirmed that the temperature variability, measured inside the isolator, does not have any effect on the radiochemical purity of radiopharmaceutical products until the expiry time. However, as required in the field of quality assurance, an assessment of the impact that conditions, other than those required by the manufacturer have on the radiopharmaceuticals for human use, is needed. 

## Figures and Tables

**Table 1:** Freeze-dried kits analyzed and the temperature required by the manufacturer for the storage of lyophilized kits and radiopharmaceutical preparations.

Name	Radiopharmaceuticals	Kit Storage Conditions ^1^	Radiopharmaceuticals Storage Conditions ^1^
Ceretec (GE Healthcare)	[^99m^Tc]Tc-HMPAO	<25 °C (1 year)	<25 °C (30 min)
MAASOL (GE Healthcare)	[^99m^Tc]Tc-albumin colloid	2–8 °C (2 year)	<25 °C (6 h)
Nanocoll (GE Healthcare)	[^99m^Tc]Tc-nanocolloid	2–8 °C (1 year)	<25 °C (6 h)
Renocis (IBA molecular)	[^99m^Tc]Tc-DMSA	2–8 °C (1 year)	15–25 °C (8h)
Technemibi (Mallinckrodt)	[^99m^Tc]Tc-MIBI	<25 °C (2 year)	<25 °C (10 h)
Technescan HDP (Mallinckrodt)	[^99m^Tc]Tc-HDP	<25 °C (2 year)	<25 °C (8 h)
Technescan MAG3 (Mallinckrodt)	[^99m^Tc]Tc-MAG3	2–8 °C (1 year)	<25 °C (8 h)

^1^ The temperature and time values of storage have been obtained from the most recently approved package inserts.

**Table 2:** Summary of the experimental conditions. We chose to use, for each preparation, the maximum activity and the maximum volume allowed by the manufacturer.

Compound	Synthesis	Quality Control System	Quality Control Time
Ceretec (GE Healthcare) [^99m^Tc]Tc-HMPAO *N* = 24	Volume: 5 mL Activity: 1.10 GBq ^1^ Incubation Time: 30 s Package leaflet: 03/2016	TLC-SA Methylethylketone (MEK)	0 min, 15 min, 30 min
		TLC-SA physiological solution	
MAASOL (GE Healthcare) [^99m^Tc] Tc-albumin colloid *N* = 24	Volume: 8 mL Activity: 2.96 GBq Incubation Time: 5 min Package leaflet: 03/2015	ITLC-SG Methanol:water (85:15, *v*:*v*)	0 h, 3 h, 6 h
Nanocoll (GE Healthcare) [^99m^Tc]Tc-nanocolloid *N* = 80	Volume: 5 mL Activity: 5.55 GBq Incubation Time: 30 min Package leaflet: 11/2015	TLC-SA Methanol:water (85:15, *v*:*v*)	0 h, 3 h, 6 h
Renocis (IBA molecular) [^99m^Tc]Tc-DMSA *N* = 8	Volume: 6 mL Activity: 3.70 GBq Incubation Time: 10 min Package leaflet: 05/2011	Whatman n.1 MEK	0 h, 4 h, 8 h
Technemibi (Mallinckrodt) [^99m^Tc]Tc-MIBI *N* = 24	Volume: 3 mL Activity: 11.10 GBq Incubation Time: 10 min, 100 °C Package leaflet: 10/2014	Baker flex aluminum oxide Ethanol >95%	0 h, 5 h, 10 h
Technescan HDP (Mallinckrodt) [^99m^Tc]Tc-HDP *N* = 60	Volume: 10 mL Activity: 11.10 GBq Incubation Time: 30 s Package leaflet: 08/2017	TLC-SG 13.6% sodium acetate	0 h, 4 h, 8 h
		TLC-SG MEK	
Technescan MAG3 (Mallinckrodt) [^99m^Tc]Tc-MAG3 *N* = 24	Volume: 10 mL Activity: 2.96 GBq ^2^ Time: 10 min, 120 °C Package leaflet: 02/2017	RP-18 54/45/1 (saline/methanol/glacial acetic acid)	0 h, 4 h, 8 h

^1^ Fresh sodium pertechnetate (not older than 2 h) obtained by generator eluted not more than 24 h before. ^2^ Fresh sodium pertechnetate obtained by generator (generator not older than 1 week from manufacturing) eluted not more than 24 h before. Where not specified, the incubation temperature during the synthesis, inside the isolator, was detected by our measurement. Finally, the production process of ^99m^Tc-radiopharmaceuticals is constantly subjected to the Fill average test.

**Table 3:** Average and standard error of temperature detected on the work surface inside the isolator.

Day ^1^	Temperature °C (8:00–12:00)	Temperature °C (12:00–18:00)
Monday	29.0 ± 1.4	30.0 ± 1.4
Tuesday	30.0 ± 1.4	30.0 ± 1.4
Wednesday	30.0 ± 1.4	29.0 ± 1.4
Thursday	30.0 ± 1.4	31.0 ± 1.4
Friday	29.0 ± 1.4	30.0 ± 1.4
Saturday/Sunday	23.0 ± 1.4	23.0 ± 1.4

^1^ The peak of activity (production and quality control of radiopharmaceuticals) is from Monday to Friday from 8:00 to 12:00 a.m.; Saturday and Sunday the Nuclear Medicine Unit is closed, therefore we consider 23 °C as the standard temperature during the non-activity period (isolator-off). The weekly radiopharmaceuticals production program is standardized and is repeated with minimal variations*.*

**Table 4:** RCP values expressed as % media ± SD and temperature values expressed as average ± standard error.

Radiopharmaceuticals	RCP-1 (%) ^1^	RCP-2 (%) ^2^	RCP-3 (%) ^3^	RCP (%) Expected Value
Ceretec (GE Healthcare) [^99m^Tc]Tc-HMPAO *N* = 24	98.1 (±1.0) 31.0 °C (±0.5)	94.8 (±1.1) 31.0 °C (±0.5)	94.5 (±2.1) 31.0 °C (±0.5)	≥80%
MAASOL (GE Healthcare) [^99m^Tc]Tc-albumin colloid *N* = 24	100.0 (±0.0) 32.0 °C (±0.5)	100.0 (±0.0) 32.0 °C (±0.5)	100.0 (±0.0) 30.0 °C (±0.5)	≥95%
Nanocoll (GE Healthcare) [^99m^Tc]Tc-nanocolloid *N* = 80	99.10 (±0.09) 29.0 °C (±0.3)	100.0 (±0.0) 29.0 °C (±0.3)	99.3 (±1.2) 29.0 °C (±0.3)	≥95%
Renocis (IBA molecular) [^99m^Tc]Tc-DMSA *N* = 8	100.0 (±0.9) 33.0 °C (±0.9)	100.0 (±0.9) 29.0 °C (±0.9)	100.0 (±0.9) 29.0 °C (±0.9)	≥95%
Technemibi (Mallinckrodt) [^99m^Tc]Tc-MIBI *N* = 24	98.4 (±0.2) 29.0 °C (±0.5)	99.1 (±0.7) 29.0 °C (±0.5)	98.9 (±0.8) 29.0 °C (±0.5)	≥94%
Technescan HDP (Mallinckrodt) [^99m^Tc]Tc-HDP *N* = 60	100.0 (±1.2) 33.0 °C (±0.3)	98.8 (±1.6) 33.0 °C (±0.3)	98.9 (±1.8) 31.0 °C (±0.3)	≥95%
Technescan MAG3 (Mallinckrodt) [^99m^Tc]Tc-MAG3 *N* = 24	100.0 (±0.0) 30.0 °C (±0.5)	99.5 (±0.5) 30.0 °C (±0.5)	99.4 (±0.5) 29.0 °C (±0.5)	≥94%

^1^ RCP-1 = value at the end of the labelling and temperature at the time of test; ^2^ RCP-2 = value determined in the middle between the labelling and the expiry time and temperature at the time of test; ^3^ RCP-3 = value determined at the expiry time and temperature at the time of test; expected value is the standard value required by the manufacturer for administration to patients.
